# Supplementary material for: Character of Electronic States in the Transport Gap of Molecules on Surfaces
Source: ACS Nano. 2023 Jun 30;17(14):13176–84. doi: 10.1021/acsnano.2c12447 (PMC10373518; doi:10.1021/acsnano.2c12447)
Supplement: Supplementary file 1 — nn2c12447_si_001.pdf [file nn2c12447_si_001.pdf]

Supporting Information – Character of electronic states in the transport gap of molecules on surfaces

Author list

Abhishek Grewal[†], Christopher C. Leon[†], Klaus Kuhnke[*,†], Klaus Kern[†,‡], Olle Gunnarsson[*,†]

Affiliations

[†]Max-Planck-Institut für Festkörperforschung, Heisenbergstraße 1, Stuttgart, 70569, Germany

[‡]Institut de Physique, École Polytechnique Fédérale de Lausanne, Lausanne, 1015, Switzerland

[*]Corresponding authors. Emails: k.kuhnke@fkf.mpg.de; o.gunnarsson@fkf.mpg.de

Simple model with one molecular level

To improve the understanding of gap states, we first consider a very simple tight-binding model where the molecule has just one (HOMO) level, and the substrate and the molecule are described in the Anderson impurity model[1]

$$H_0^{\mathrm{SH}} = \sum_{\sigma} \varepsilon^{\mathrm{H}} n_\sigma + \sum_{\mathbf{k}\sigma} \varepsilon_{\mathbf{k}}^{\mathrm{S}} n_{\mathbf{k}\sigma}^{\mathrm{S}} + \sum_{\mathbf{k}\sigma} V^{\mathrm{SH}} \left[c_\sigma^\dagger \left(c^{\mathrm{S}}\right)_{\mathbf{k}\sigma} + \left(c^{\mathrm{S}}\right)_{\mathbf{k}\sigma}^\dagger c_\sigma\right]. \tag{1}$$

Here, the first term describes the molecular HOMO level with the energy ε^{H} and occupation number n_σ for electrons with spin σ. The second term describes the substrate levels with quantum numbers $\mathbf{k}$ and energies $\varepsilon_{\mathbf{k}}^{\mathrm{S}}$. The third term describes the hopping between the substrate and the HOMO level with the hopping integral V^{SH}. The corresponding annihilation operators are $\left(c^{\mathrm{S}}\right)_{\mathbf{k}\sigma}$ and c_σ. The substrate has a semi-elliptic density of states (DOS), $\rho(\varepsilon)$, with the width $2B$

$$\rho(\varepsilon) = \sum_{\mathbf{k}} \delta\left(\varepsilon_{\mathbf{k}}^{\mathrm{S}} - \varepsilon\right) = \frac{2}{\pi B^2}\sqrt{B^2 - \varepsilon^2}\, \Theta\left(B^2 - \varepsilon^2\right), \tag{2}$$

where $\Theta(x) = 1$ for $x > 0$ and zero otherwise. The tip is described by the Hamiltonian

$$H_0^{\mathrm{T}} = \sum_{\mathbf{k}\sigma} \varepsilon_{\mathbf{k}}^{\mathrm{T}} n_{\mathbf{k}\sigma}^{\mathrm{T}}, \tag{3}$$

where $\varepsilon_{\mathbf{k}}^{\mathrm{T}}$ describes the tip energies. The tip also has a semi-elliptic DOS, but it is displaced in energy by the bias, U_{bias} $(-B \le U_{\mathrm{bias}} \le 0)$. We treat the coupling between the molecule and the tip as a perturbation

$$V(t) = e^{\delta t} V_0 \sum_{\mathbf{k}\sigma} \left[\left(c^{\mathrm{T}}\right)_{\mathbf{k}\sigma}^\dagger c_\sigma + c_\sigma^\dagger \left(c^{\mathrm{T}}\right)_{\mathbf{k}\sigma}\right], \tag{4}$$

where $\left(c^{\mathrm{T}}\right)_{\mathbf{k}\sigma}$ annihilates an electron on the tip. The perturbation $V(t)$ is turned on adiabatically at a time $t = t_0 \to -\infty$ with the time dependence $\exp(\delta t)$, where $\delta > 0$ and $\delta \to 0$ so that $|t_0|\delta \gg 1$. The full Hamiltonian is

$$H = H^{\mathrm{SH}} + H^{\mathrm{tip}} + V(t). \tag{5}$$

We first solve the Anderson impurity model. The local DOS, $\rho^{\mathrm{HOMO}}(\varepsilon)$, on the HOMO level is[1]

$$\rho^{\mathrm{HOMO}}(\varepsilon) = \frac{1}{\pi} \mathrm{Im} \frac{1}{\varepsilon - \varepsilon^{\mathrm{H}} - \Lambda(\varepsilon) - i\Delta(\varepsilon)} \tag{6}$$

where

$$\Delta(\varepsilon) = \frac{2\left(V^{\mathrm{SH}}\right)^2}{B^2} \sqrt{B^2 - \varepsilon^2}\, \Theta\left(B^2 - \varepsilon^2\right) \tag{7}$$

and

$$\Lambda(\varepsilon) = \frac{2\left(V^{\mathrm{SH}}\right)^2}{\pi B^2} \begin{cases} \varepsilon, & \text{if } |\varepsilon| \leq B, \\ \varepsilon + \sqrt{\varepsilon^2 - B^2}, & \text{if } \varepsilon < -B, \\ \varepsilon - \sqrt{\varepsilon^2 - B^2}, & \text{if } \varepsilon > B. \end{cases} \tag{8}$$

We rewrite the Anderson Hamiltonian, H_0^{SH}, in terms of the eigenstates as

$$H_0^{\mathrm{SH}} = \sum_{i\sigma} \varepsilon_i^{\mathrm{SH}} n_{i\sigma}^{\mathrm{SH}}, \tag{9}$$

where $\varepsilon_i^{\mathrm{SH}}$ are the eigenvalues of H_0^{SH} and $n_{i\sigma}^{\mathrm{SH}}$ the corresponding number operators. We now introduce the hopping between the Anderson impurity and the tip in terms of the new operators c^{SH}

$$V(t) = e^{\delta t} \sum_{ij\sigma} W_i [\left(c^{\mathrm{SH}}\right)_{i\sigma}^{\dagger} \left(c^{\mathrm{T}}\right)_{j\sigma} + \left(c^{\mathrm{T}}\right)_{j\sigma}^{\dagger} \left(c^{\mathrm{SH}}\right)_{i\sigma}], \tag{10}$$

where W_i is expressed in terms of V_0 and the solutions $|i\rangle$ of the Anderson model

$$|i\rangle = \left(a^i c^{\dagger} + \sum_{\mathbf{k}} a_{\mathbf{k}}^i c_{\mathbf{k}}^{\dagger} \right) |\mathrm{vacuum}\rangle. \tag{11}$$

Then

$$W_i = a^i V_0. \tag{12}$$

We then also have that

$$\rho^{\mathrm{HOMO}}(\varepsilon) = \sum_i \left|a^i\right|^2 \delta\left(\varepsilon - \varepsilon_i^{\mathrm{SH}}\right) \equiv |a(\varepsilon)|^2 \rho(\epsilon), \tag{13}$$

since a^i only depends on $\varepsilon_i^{\mathrm{SH}}$.

We consider an initial state $|a\rangle$, where both the Anderson model and the tip are filled up to their Fermi levels at $\varepsilon_F^{\mathrm{S}} = 0$ and $\varepsilon_F^{\mathrm{T}} = U_{\mathrm{bias}}$, respectively. The corresponding energy, $E_a = 0$, we use as energy zero. In a final state, there is a hole in the Anderson model at energy $E_i \leq 0$ and an electron

on the tip with energy $E_j \geq U_{\text{bias}}$. The first order perturbation theory transition amplitude between the initial state $|a\rangle$ and a state $|ij\rangle$ is given by

$$\langle ij|U^{(1)}|a\rangle = \frac{1}{i\hbar}\int_{\infty}^{t} \mathrm{d}\tau e^{[-i(E_j-E_i)(t-\tau)/\hbar+\delta\tau]}\, W_i = \frac{e^{\delta t}}{E_j - E_i + i\delta\hbar} W_i, \tag{14}$$

We then have the probability

$$\left|\langle ij|U^{(1)}|a\rangle\right|^2 = |W_i|^2 \left|\frac{1}{E_j - E_i + i\delta\hbar}\right|^2 e^{2\delta t} \tag{15}$$

$$\rightarrow \frac{\pi}{\delta\hbar}|W_i|^2 e^{2\delta t}\delta(E_i - E_j) \tag{16}$$

We then perform the sums over i, j, and spin σ

$$\sum_{ij\sigma}\left|\langle ij|U^{(1)}|a\rangle\right|^2 = \frac{2\pi}{\delta\hbar}\sum_i |W_i|^2\, \rho(E_i - U_{\text{bias}})e^{2\delta t} \tag{17}$$

$$= \frac{2\pi V_0^2}{\delta\hbar}\sum_i \left|a^i\right|^2 \rho(E_i - U_{\text{bias}})e^{2\delta t}$$

$$= \frac{2\pi V_0^2}{\delta\hbar}\int_{U_{\text{bias}}}^{0} \mathrm{d}\varepsilon\, \rho^{\text{HOMO}}(\varepsilon)\rho(\varepsilon - U_{\text{bias}})e^{2\delta t}$$

The current is obtained by taking the derivative with respect to t. For $t = 0$ we obtain

$$\frac{4\pi V_0^2}{\hbar}\int_{U_{\text{bias}}}^{0} \mathrm{d}\varepsilon \rho^{\text{HOMO}}(\varepsilon)\rho(\varepsilon - U_{\text{bias}}). \tag{18}$$

The quantity is shown in Figure 1 in the main text, normalized to its value at $U_{\text{bias}} = -2$ V. It represents a dimensionless rate of transfer of electrons at $t = 0$.

In this discussion, we have neglected the Coulomb interaction. We could introduce a new Hamiltonian for the HOMO level with the interaction U

$$H^{\text{M}} = \tilde{\varepsilon}^{\text{H}}\sum_\sigma n_\sigma + U n_\uparrow n_\downarrow. \tag{19}$$

Making the transformation $\varepsilon^{\text{H}} = \tilde{\varepsilon}^{\text{H}} + U$ we obtain

$$H^{\text{M}} = \varepsilon^{\text{H}}\sum_\sigma n_\sigma + U(1 - n_\uparrow)(1 - n_\downarrow) - U. \tag{20}$$

If the HOMO is well below the Fermi energy of the substrate, the state with no HOMO electron is not very important and can be neglected. Then the Coulomb energy is not very important, except for

renormalization of the effective HOMO energy. However, if the HOMO is close to the substrate Fermi energy, this is not true, and the Coulomb energy substantially changes the physics of the problem. The same is true if we include a LUMO in the problem. The Coulomb interaction then leads to excitons which would otherwise be neglected.

In our tight-binding model above, we assumed that all hopping from the substrate to the tip goes via the HOMO state and that there is no direct hopping from the substrate state to the tip states. The $1d$ model in the following section can be solved without introducing a basis set and thereby without assumptions about which hopping integrals can be neglected. We then show that assumptions similar to those above do not change the results very much.

$\mathbf{1d}$ model

We now give some details of the one-dimensional ($1d$) model in the main text. We introduce a substrate potential

$$V_{\text{substr}}(z) = \begin{cases} \infty, & \text{for } z < -64 \\ -10.4, & \text{for } -64 \leq z \leq 0 \\ 0, & \text{for } z > 0 \end{cases} \tag{21}$$

and a molecular potential

$$V_{\text{mol}} = V_0[\delta(z - z_0) + \delta(z - z_0 - d)], \tag{22}$$

where $z_0 = 10$, $d = 3$ and V_0 is chosen so that the levels of the free molecule are at -7.1 eV and -3.5 eV. All lengths are measured in Bohr radii, a_0, and all energies in eV. We then solve the model

$$H = H_0 + V_{\text{substr}} + V_{\text{mol}} \tag{23}$$

exactly, where H_0 is the kinetic energy operator. These solutions are shown in Figure 1 below. We consider one energy below the HOMO, two energies between the HOMO and the LUMO, and one energy above the LUMO.

In the main text, we considered a model of PtPc on a NaCl film on Au. These solutions are described by a basis set, where we include Au states, valence and conduction states of NaCl, and bound states of PtPc. However, we do not introduce additional basis states to describe states in the gaps of NaCl and PtPc. Since we know the exact solutions of the $1d$ model, we can test this

approximation by expanding the exact solutions in the solutions of the free molecule in the model above

$$(H_0 + V_{\mathrm{mol}})|i\rangle = E_i|i\rangle, \qquad i = 1, 2. \tag{24}$$

where $|i\rangle,\ i = 1,\ 2$, are the bonding and antibonding solutions of the molecule. We then calculate

$$|K\rangle - \sum_{i=1}^{2} \langle i|K\rangle |i\rangle, \tag{25}$$

where $|K\rangle$ is a solution of the model.

In Figure 1 below, we subtract the part of the solutions that can be expanded in the HOMO and LUMO and show the remainder. The main point is that this remainder is minimal in the range of the molecule ($10 \le z \le 13$) and that using the HOMO and LUMO states as the only basis functions in this range is a good approximation.

Tight-binding model

We use a model consisting of a three-layer film on a Au(111) substrate. The NaCl film contains $9 \times 9 \times 4 = 324$ atoms per layer. We use three different clusters representing the Au substrate and average the results. The three Au clusters have four, six or eight layers with 1020, 780 and 572 Au atoms per layer, respectively. In total there are then 648 or 972 atoms in the NaCl film and 4080, 4680 or 4576 atoms in the Au substrate. We impose periodic boundary conditions parallel to the surface for the NaCl slab and for the Au slabs. All hopping integrals are constructed according to the rules of Harrison,[2,3] including $s - d$[3] and $p - d$ hopping. The NaCl(100) and Au(111) surfaces are non-commensurate. We place the central Na atom on top of the central Au atom in the neighboring NaCl and Au layers.

To describe the Au substrate, we use the lattice parameter $a_{\mathrm{Au}} = 4.07$ Å.[4] We use the Harrison level energies $\varepsilon_{6s} = -6.98$ eV and $\varepsilon_{5d} = -17.78$ eV as a starting point and add a $6p$ level at 5 eV above the $4s$-level. We then shift the $5d$-level relative to the $6s$ and $6p$ so that the top of the $5d$ band is placed at 1.7 eV below the Fermi energy.[5] Finally all energies are shifted so that the Fermi energy is at zero. These parameters are summarized in Table 1.

To describe the NaCl film, we followed our earlier work[6] and chose parameters such that the conduction band has mainly Cl $4s$ character[6,7]. For this purpose, we replace the Cl $3s$ level by a $4s$ level, which has been strongly lowered by the Madelung potential, while the Na levels are shifted strongly upwards. We adjust the Harrison parameters so that the experimental band gap (8.5 eV[8]) is reproduced for bulk NaCl. According to the calculations[9] using the GW method[10], the top of the valence band is 5 eV below the Fermi energy. We then shift all the NaCl energies relative to the Au energies correspondingly. The resulting parameters are summarized in Table 1. The calculations were performed using the lattice parameter $a_{\mathrm{NaCl}} = 5.54$ Å.[11]

We use the calculated separation $d_{\mathrm{Au-NaCl}} = 3.12$ Å between the Au surface and the NaCl film.[11] Since the NaCl film and the substrate are incommensurate, several Au atoms can have similar distances to a given NaCl atom, and the nearest neighbors are poorly defined. We then use a smooth distance dependent cut off of the Harrison prescription for the hopping between the substrate and the film. Thus the Harrison prescription for these hopping integrals is multiplied by a factor

$$\exp\left(-\frac{(d - d_{\mathrm{Au}} - d_{\mathrm{NaCl}})^2}{\lambda_{\mathrm{SB}}^2}\right), \tag{26}$$

where d is the distance between an Au atom and a NaCl atom at the Au-NaCl interface. Here λ_{SB} is chosen such that summing these factors over all the Au neighbors of a NaCl atom and averaged over the NaCl atoms in the innermost layer adds up to four. Then the innermost NaCl atoms effectively couple to four Au atoms.

We study the adsorbed molecules platinum phthalocyanine (PtPc) and magnesium phthalocyanine (MgPc). The coordinates of PtPc are obtained from a density functional calculation. The same coordinates are used for MgPc. The tight-binding parameters are obtained from Harrison[2] and are given in Table 1. For the H atoms, we include the $1s$ level at the energy -13.6 eV (not given by Harrison). Guided by Miwa *et al.*,[12] we use the separation 3.4 Å between the molecules and the NaCl film both for PtPc and MgPc. MgPc is adsorbed on top of a Cl atom, while PtPc is adsorbed on top of a Na atom. For PtPc, the four "arms" of the molecule are along the NaCl(100) directions, while for MgPc, they are close to the NaCl(110) directions with a small deviation of 8° included in the calculation.[12] Again a λ_{BM} is chosen so that, on average, from each atom in the molecule there is

effectively hopping to four atoms in the NaCl buffer. The Au slab breaks the four-fold symmetry of PtPc and MgPc which has been reintroduced in the plots.

For the PtPc molecule, these parameters incorrectly put a σ-orbital below the HOMO. We, therefore, shift this orbital upwards by 3.2 eV. The parameters in Table 1 lead to a too small gap. We then shift the occupied levels downwards so that the HOMO of the free molecule is at -1.42 eV and the unoccupied levels upward so that the LUMO is 1.69 eV. After the interactions with the substrate and NaCl are included, the HOMO is then at -1.3 eV and the LUMO at 1.7 eV ($E_F = 0$), in agreement with experiment.

Experimental characterization of PtPc atop 3 ML NaCl/Au(111)

Figure 2A below shows an overview image of the area where experimental results shown in Figure 5 in the main text are obtained. The topographic height of NaCl as determined from fitting an error function to the line profile is 4.74±0.01 Å (shown in Figure 2B). The in-gap image of PtPc atop 3 ML NaCl shows a sample voltage dependence and has a topographic height of $\approx 0.6 - 1.2$ Å for sample voltage of -0.2 to -1 V. Figure 2C shows the STM topography image of HOMO orbitals of the PtPc atop 3 ML NaCl on Au(111). The higher set point used (20 pA) allows simultaneous imaging of the Cl ions of NaCl(100) lattice. The white dashed lines in Figure 2C mark the Na ion rows of NaCl(100). The intersection of two Na ion rows overlapping with the center of the PtPc HOMO topography indicates that PtPc is adsorbed atop the Na ion. We emphasize that the experimental results for PtPc are obtained using a metallic Au tip which is distinct from the carbon monoxide (CO) decorated tip employed by Miwa *et al.*[12]

Comparing in-gap maps in constant current and constant height modes

In the main text, we compare the calculations to constant height scans in Figure 4 and to constant current scans in Figure 5. In the following we show that there is no qualitative difference between in-gap images in constant height and constant current mode. Note that in the vacuum tail of the electronic wave function probed by STM, the current depends exponentially on the distance, which provides the basis for the functional relation between the different types of maps. Plotting both maps

with the same color scale overemphasizes the contrast in constant height maps and lets weak features often disappear within experimental noise. In Figure 4 below we compare calculations (A, B) with constant height maps (C, D) and constant current maps (E, F). We find that by using the same linear color scales, the constant height maps show a closer resemblance to theory because the local density of states in the calculation is also evaluated at a constant height above the molecule. However, the observed “cross” shape of the in-gap feature is the same in all cases.

d*I*/d*V* spectroscopy in the transport gap

In Figure 5 below, we compare dI/dV spectra obtained by theory (A) with experimental data (B, C). The dynamics of the STM data is limited by instrumental noise typically to about 1 pA/V in spectra that allow to capture in-gap features together with the signal of the frontier orbitals. In Figure 5B and C, we use the numerical derivative of the current signal in order to avoid the zero offset, which is typically present in data from a lock-in amplifier. By doing this, we can identify in Figure 5B a value of 2pA/V around zero sample voltage which can be recognized as a true signal indicated by the reduced scattering of data points on the logarithmic scale. In Figure 5C, this signal is even more pronounced. The in-gap dI/dV signal is about 2-3 orders of magnitude below the signal at the top of the HOMO and thus close to the result of the calculation. The pronounced step in Figure 5C near 0 eV may be due to the additional electronic density of states from the Au-NaCl interface state, which has a sharp onset around -0.25 eV.[13] At positive voltages, the calculation exhibits the step around +1 eV, which we attribute to the same interface state which is, however, shifted due to the employed vacuum level alignment of energies which may not agree with the experimental alignment between substrate and molecular states.

References

(1) Anderson, P. W. Localized Magnetic States in Metals. *Phys. Rev.* **1961**, *124* (1), 41–53. https://doi.org/10.1103/PhysRev.124.41.
(2) Harrison, W. *Elementary Electronic Structure*; World Scientific Publishing: Singapore, 1999. https://doi.org/10.1142/4121.
(3) Harrison, W. A. *Electronic Structure and the Properties of Solids: The Physics of the Chemical Bond*; Freeman, 1980.
(4) Davey, W. P. Precision Measurements of the Lattice Constants of Twelve Common Metals. *Phys. Rev.* **1925**, *25* (6), 753–761. https://doi.org/10.1103/PhysRev.25.753.

(5) Sheverdyaeva, P. M.; Requist, R.; Moras, P.; Mahatha, S. K.; Papagno, M.; Ferrari, L.; Tosatti, E.; Carbone, C. Energy-Momentum Mapping of d-Derived Au(111) States in a Thin Film. *Phys. Rev. B* **2016**, *93* (3), 035113. https://doi.org/10.1103/PhysRevB.93.035113.
(6) Leon, C. C.; Grewal, A.; Kuhnke, K.; Kern, K.; Gunnarsson, O. Anionic Character of the Conduction Band of Sodium Chloride. *Nat Commun* **2022**, *13* (1), 981. https://doi.org/10.1038/s41467-022-28392-8.
(7) de Boer, P. K.; de Groot, R. A. The Origin of the Conduction Band in Table Salt. *American Journal of Physics* **1999**, *67* (5), 443–445. https://doi.org/10.1119/1.19282.
(8) Poole, R. T.; Jenkin, J. G.; Liesegang, J.; Leckey, R. C. G. Electronic Band Structure of the Alkali Halides. I. Experimental Parameters. *Phys. Rev. B* **1975**, *11* (12), 5179–5189. https://doi.org/10.1103/PhysRevB.11.5179.
(9) Wang, S.; Kharche, N.; Costa Girão, E.; Feng, X.; Müllen, K.; Meunier, V.; Fasel, R.; Ruffieux, P. Quantum Dots in Graphene Nanoribbons. *Nano Lett.* **2017**, *17* (7), 4277–4283. https://doi.org/10.1021/acs.nanolett.7b01244.
(10) Hedin, L. New Method for Calculating the One-Particle Green's Function with Application to the Electron-Gas Problem. *Phys. Rev.* **1965**, *139* (3A), A796–A823. https://doi.org/10.1103/PhysRev.139.A796.
(11) Chen, H.-Y. T.; Pacchioni, G. Properties of Two-Dimensional Insulators: A DFT Study of Co Adsorption on NaCl and MgO Ultrathin Films. *Phys. Chem. Chem. Phys.* **2014**, *16* (39), 21838–21845. https://doi.org/10.1039/C4CP03470H.
(12) Miwa, K.; Imada, H.; Kawahara, S.; Kim, Y. Effects of Molecule-Insulator Interaction on Geometric Property of a Single Phthalocyanine Molecule Adsorbed on an Ultrathin NaCl Film. *Phys. Rev. B* **2016**, *93* (16), 165419. https://doi.org/10.1103/PhysRevB.93.165419.
(13) Lauwaet, K.; Schouteden, K.; Janssens, E.; Haesendonck, C. V.; Lievens, P. Dependence of the NaCl/Au(111) Interface State on the Thickness of the NaCl Layer. *J. Phys.: Condens. Matter* **2012**, *24* (47), 475507. https://doi.org/10/gf98z8.

Table 1 – Level energies used for PtPc, MgPc, NaCl, and Au after shifts described in the text. The Fermi energy is put at zero.

Element	s	p	d
Au $(6s, 6p, 5d)$	4.1	9.1	−3.7
Na $(3s, 3p)$	12.8	16.8	–
Cl $(4s, 3p)$	10.2	−5.0	–
C $(2s, 2p)$	−19.38	−11.07	–
N $(2s, 2p)$	−26.22	13.84	–
Pt $(6s, 5d)$	−6.85	–	−16.47
Mg $(3s, 3p)$	−6.89	−3.79	–
H $(1s)$	−13.61	–	–

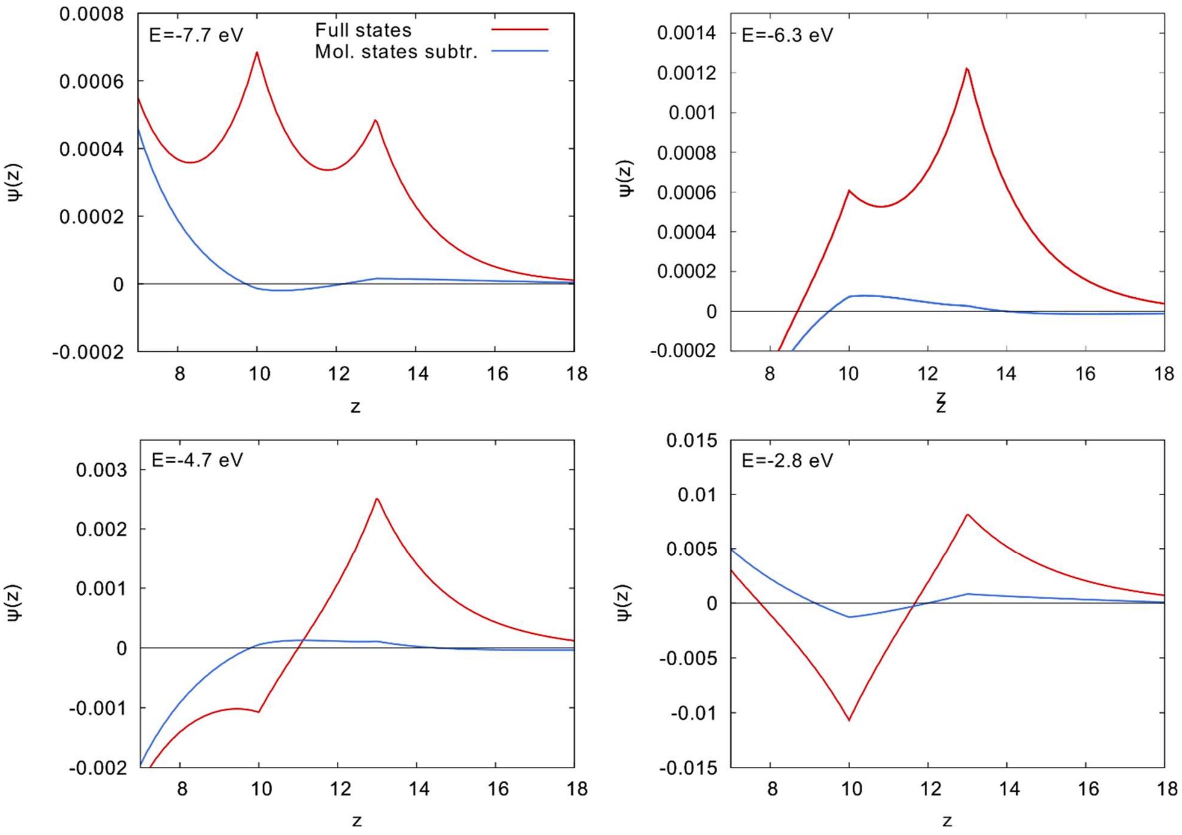

Figure 1 – Solutions of the $1d$ model for different energies in the range of the molecule. We also show the rest after the free molecule solutions have been subtracted [see Eq. 25]. The rest is very small in the range of the molecule $(10 \leq z \leq 13)$, meaning that the two solutions provide an efficient basis set in this range and for these energies. As the energy is increased, the solution changes from bonding to antibonding character. Below the HOMO and above the LUMO the solution has a larger weight on the innermost atom while between these energies it has most weight on the outermost atom. This can easily be understood by studying the interference between the terms in an expansion in the HOMO and LUMO.

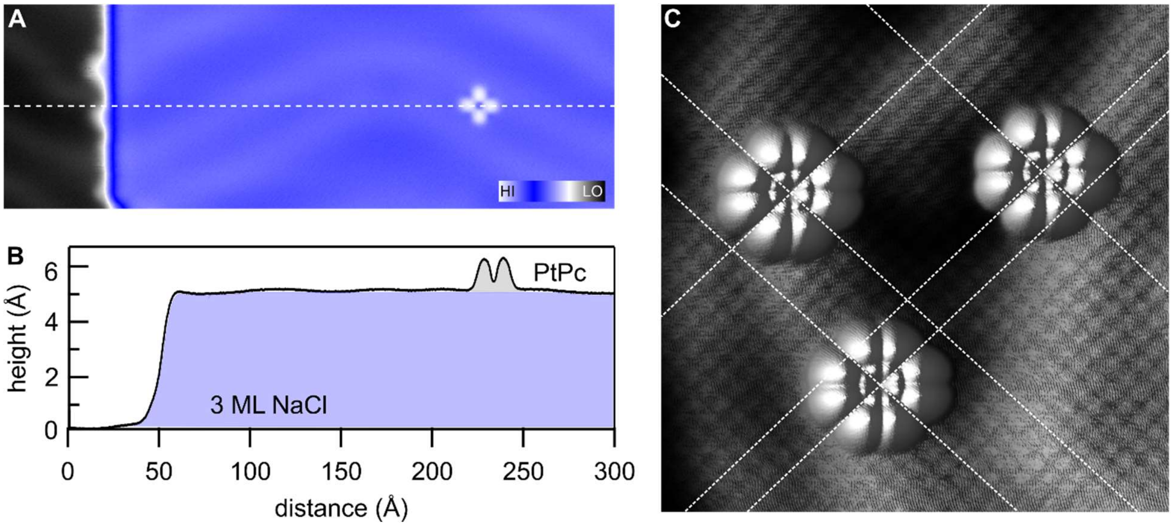

Figure 2 – Overview image of STM topography for PtPc adsorbed atop 3 ML NaCl on Au(111). (A) Overview image of PtPc atop 3 ML NaCl on Au(111) using set point: 1 pA, -1 V ($300 \times 100\,\text{Å}^2$). (B) Height profile of the dashed line marked in A (C) STM topography image showing the HOMO of the PtPc and Cl ions of the NaCl(100) lattice using set point: 20 pA, -1.23 V ($100 \times 100\,\text{Å}^2$). The white dashed lines mark the position of rows of Na ions.

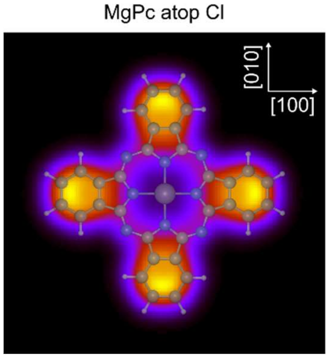

Figure 3 – Theoretical result for MgPc adsorbed atop Cl as in Ref. 12 but orientated along (010) axis of the NaCl with the molecular ball-and-stick model overlaid. The orientation is similar to that of PtPc or H_2Pc. Size: $20 \times 20\,\text{Å}^2$.

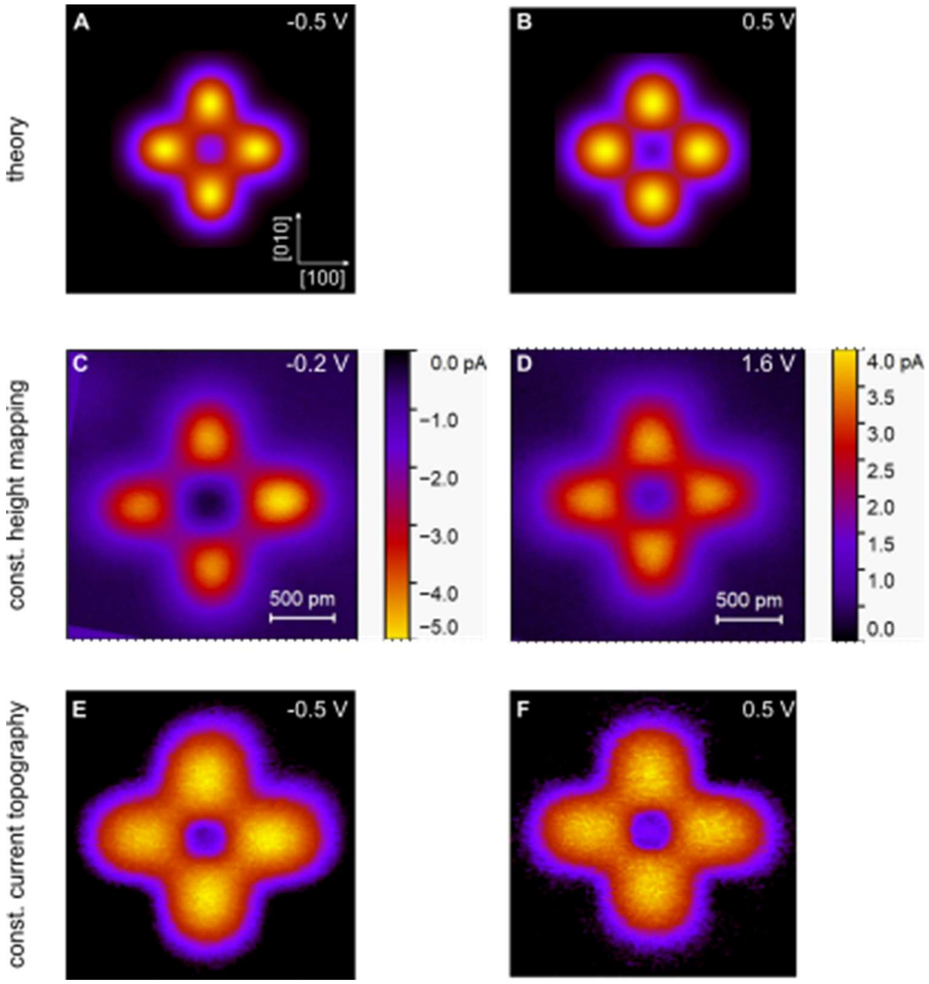

Figure 4 – Maps of PtPc molecules in the transport gap for sample voltages given in the top right corner of each panel. (A,B) theoretical results. (C,D) Experimental constant height maps of a molecule with current scales given on the right of each panel. (E,F) Experimental constant current maps on a different molecule. The length scale for all panels is given in (C) and (D).

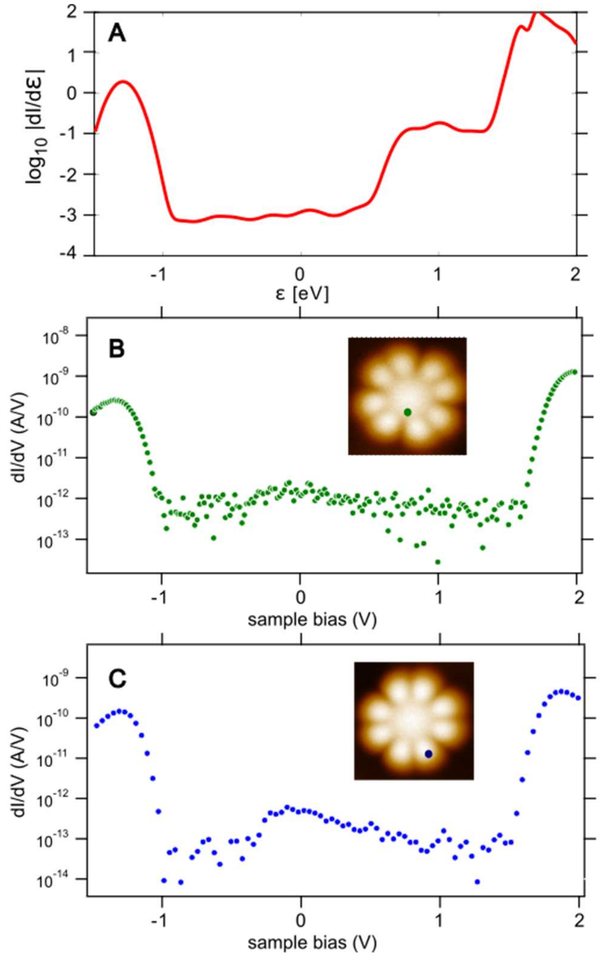

Figure 5 – (A) Differential conductance from theory for a PtPc molecule on 3 monolayers of NaCl averaged over the entire molecule. (B,C) STM current signal numerically differentiated with respect to bias voltage. The spectra are local measurements on the positions marked by the dot on the respective inset.
